# An Account of the Nature and Medicinal Virtues of the Principal Mineral Waters of Great Britain and Ireland, and Those Most in Repute on the Continent. To Which Are Prefixed, Directions for Impregnating Water with Fixed Air, in Order to Communicate to It the Peculiar Virtues of Pyrmont Water, and Other Mineral Waters of a Similar Nature; Extracted from Dr. Priestley's Experiments on Air. With an Appendix, Containing a Description of Dr. Nooth's Apparatus, with the Improvements Made in It by Others; and a Method of Impregnating Water with Sulphureous Air, so as to Imitate the Aix-La-Chapelle and Other Sulphureous Waters

**Published:** 1781-11

**Authors:** 


V. *An account of the nature and medicinal virtues of the principal mineral waters of Great Britain and Ireland, and thoſe moſt in repute on the continent. To which are prefixed, directions for impregnating water with fixed air, in order to communicate to it the peculiar virtues of Pyrmont water, and other mineral waters of a ſimilar nature; extracted from Dr. Prieſtley's experiments on air. With an appendix, containing a deſcription of Dr. Nooth's apparatus, with the improvements made in it by others; and a method of impregnating water with ſulphureous air, ſo as to imitate the Aix-la-Chapelle and other ſulphureous waters. By* John Elliot, M. D. 8vo. Johnſon, *London*, 1781. 236 pages, with a copper plate.

IN an introduction to this work the author gives a general view of his ſubject, with ſome remarks on the medicinal properties and methods of uſing different mineral waters, which he has arranged in the form of tables, under the ſeveral heads of, 1. Chalybeate waters; 2. Chaly-

Chalybeate purging waters; 3. Sulphureous waters; 4. Sulphureous purging waters; 5. Acidulous or faline waters; 6. Saline purging waters; 7. Vitriolic waters; 8. Waters which contain an earth.—The introduction concludes with the following fhort account of the moft obvious methods of analyzing, or difcovering the nature of mineral waters.

"1. Waters are known to contain iron by their exhibiting a purple or black colour with the infufion of galls; and the quantity of iron which they contain, that is, their ftrength as chalybeate waters, is determined by the deepnefs of the colour; the quantity and ftrength of the infufion being the fame. If the iron be held in folution by fixed air, the latter will fly off on expofing the water in an open veffel, and then the iron will fall to the bottom in the form of a yellowifh or reddifh brown powder, or oker. But if the iron be held in folution by the vitriolic acid, in the form of green vitriol, or copperas, this precipitation of oker does not take place. The chalybeate water may alfo be known to be vitriolic by its auftere ftyptic tafte.

2. If, on the addition of fyrup of violets to a water, it turns to a bright green, it fhews that an alkaline falt is contained in the water, and the only alkaline ſalt ever found in mineral water is the foſſil.

When this kind of water, however, is firſt taken up from the well, the alkaline ſalt is uſually ſaturated with fixed air, and therefore does not change ſyrup of violets green. It is frequently even ſuperſaturated with fixed air, and therefore turns the ſyrup red. But the air ſoon flies off on expoſure, and then the effect is as above-mentioned; the water is alſo found ſofter than common water, and lathers better with ſoap.

But earthy and metallic ſubſtances alſo change ſyrup of violets green. To be certain therefore that it is the foſſil alkali, add a little fixed alkaline ſalt, and if no powder falls to the bottom, and the water does not become turbid, it may be concluded that it is the foſſil alkali with which the water is impregnated.

3. If on the addition of ſyrup of violets a red colour is obſerved, the water contains an acid. Thus, fixed air is an acid; and when waters which are ſtrongly impregnated with fixed air, are firſt taken up from the fountain, they are found to change ſyrup of violets red. As the air flies off, however, this redneſs diſappears; and if the water alſo contained an alkali, it will, on on the further escape of the fixed air turn green, for the reason given in the last article.

4. If water contains sea salt, oil of vitriol dropt into it will cause white noxious fumes to arise; which is the acid of the sea salt, whose place the vitriolic acid had taken in the alkaline basis of the sea salt.

If a sufficient quantity of the oil of vitriol be added, and the water be evaporated, not sea salt but a true Glauber's salt will remain behind.

Also the residuum of these waters crackles and flies when placed on a red-hot iron.

5. If a mineral water contains that kind of purging salt which the chemists call calcareous Glauber's, or the Epsom salt (the purging salts usually found in mineral waters) a solution of fixed alkali added will make the water turbid, and the earthy basis of the salt will fall to the bottom in form of a white powder.

6. Sulphureous waters are known by their smell, and by their changing silver of a reddish copper colour.

7. If water contains an earth deprived of fixed air, it may be discovered by adding fixed air to it; for then the water will become turbid, and the earth will fall to the bottom. If, on the contrary, it contains an earth supersaturated with fixed air, drawing part of it away by the air pump, or expofing the water to the air, or to warmth, will alfo precipitate the earth.

Waters in general contain feveral kinds of thefe folid matters, and therefore more than one of thefe methods are to be employed in detecting them.

8. Water may be known to contain fixed air by its fparkling on being poured from one veffel to another, by the explofion which follows on its being fhook in a phial half filled with it, and by the bubbles which arife when placed over the fire, long before it is hot enough to boil.

The folid matters contained in waters may alfo be known by evaporating the waters and examining their refiduums."

In the work itfelf the different waters are arranged alphabetically. As it is impoffible to abridge a performance of this nature, we fhall felect the following accounts of the Briftol and Malvern waters as fpecimens of the manner in which the author has treated his fubject.

"BRISTOL, in Somerfetfhire.

The fprings are known by the name of the Hot Wells.

The

The water at its origin is warm, clear, pellucid and ſparkling; and if let ſtand in a glaſs, covers its inſide with ſmall air-bubbles. It has no ſmell, and is ſoft and agreeable to the taſte. It raiſes the thermometer from about ſeventy to eighty degrees. It contains an earthy matter, which is ſuſpended by means of fixed air, together with ſea ſalt, and a ſpecies of Glauber's ſalt. The quantities of theſe latter ingredients however are very ſmall.

It has been recommended in a variety of diſorders. In conſumptions and weakneſs of the lungs; in caſes attended with hectic fever and heat (in which, among other properties, it differs from the Bath water) in uterine and other internal hæmorrhages, and in immoderate diſcharge of the menſes; in old diarrhœas and dyſenteries; in the fluor albus; in gleets; in the diabetes; and in other caſes where the ſecretions are too much increaſed, and the humours too thin; in the ſtone and gravel; in the ſtrangury; in colliquative ſweats; in ſcorbutic and ſimilar caſes; in cholics; in the gout and rheumatiſm; in loſs of appetite and indigeſtion; and in many other diſeaſes.

The uſual method of drinking the water is a glaſs or two before breakfaſt, and about five in the the afternoon. The next day three glaſſes before breakfaſt, and as many in the afternoon; and this is to be continued during the patient's ſtay at the Wells. A quarter or half an hour is allowed between each glaſs.

A courſe of theſe waters requires no preparation further than to empty the bowels by ſome gentle purge; and if heat or fever requires, to take away a few ounces of blood. Coſtiveneſs, however, ſhould be avoided during the courſe.

Externally they are uſeful in ſore and inflamed eyes; in ſcrophulous and cancerous ulcers; and in other ſimilar caſes.

This water is cooling and quenches the thirſt. It is beſt drank at the ſpring head; though it will bear carriage tolerably well."

" MALVERN, in Glouceſterſhire.

There are two noted ſprings at this place, one of them called the Holy Well, in the midway between Great and Little Malvern, the other is about a quarter of a mile from Great Malvern. But the waters are not materially different.

They are light and pleaſant chalybeates, and are remarkable for being almoſt entirely free from any earthy matter. For three quarts of the Holy Well water being evaporated, ſcarce the fourth part of a grain of ſediment was left behind.

They are recommended as excellent in diſeaſes of the ſkin; in leproſies; ſcorbutic complaints; the King's evil; glandular obſtructions; ſcald heads; old ſores; cancers, &c. They are alſo ſerviceable in inflammations and other diſeaſes of the eyes; in the gout and ſtone; in cachectic, bilious, and paralytic caſes; in old head-achs, and in female obſtructions.

The external uſe is by waſhing the part under the ſpout ſeveral times in a day; afterwards covering the part with cloths dipt in the water, which muſt be kept conſtantly moiſt. Thoſe who bathe, uſually go into the water with their linen on, and dreſs upon it wet, and it is never found to be attended with inconvenience.

The waters, when firſt drank, are apt to occaſion, in ſome, a ſlight nauſea; others they purge briſkly for ſeveral days; but they operate by urine in all.

It is adviſeable to drink freely of the waters for ſome days before they are uſed externally."

In his account of the methods of impregnating water with fixed air, the author obſerves, that Mr. Blades has made a conſiderable improvement in Dr. Nooth's apparatus, by changing the ſtopple for a glaſs cock, and by altering the middle veſſel into a form more advantageous for the impregnation.

Dr. Elliot's methods of imitating ſulphureous and other mineral waters are ſimilar to thoſe deſcribed by Profeſſor Bergman, an account of which will be found in the firſt volume * of our Journal.

